# The Perception of Allied Health Professionals on Occupational Therapy

**DOI:** 10.1155/2022/2588902

**Published:** 2022-03-09

**Authors:** Farahiyah Wan Yunus, Nuralia Fatiha Ahmad Ridhuwan, Muhammad Hibatullah Romli

**Affiliations:** ^1^Centre for Rehabilitation and Special Needs Studies, Occupational Therapy Programme, Faculty of Health Sciences, Universiti Kebangsaan Malaysia, Jalan Raja Muda Abdul Aziz, 50300 Kuala Lumpur, Malaysia; ^2^Center for Psychology and Rehabilitation of Atfal Jannah, Jalan Pinang 7, Bukit Kuang, 24000 Kemaman, Terengganu, Malaysia; ^3^Department of Rehabilitation Medicine, Faculty of Medicine and Health Sciences, Hospital Pengajar Universiti Putra Malaysia, Universiti Putra Malaysia, 43400 Serdang, Selangor, Malaysia; ^4^Malaysian Research Institute on Ageing (MyAgeing™), Universiti Putra Malaysia, 43400 Serdang, Selangor, Malaysia

## Abstract

Occupational therapy is a client-centered health profession and is a part of an interdisciplinary team. Effective interdisciplinary practice occurs when each professional understands the role of another professional in the team. This resulted in optimal referral among the professionals that can benefit the clients to receive good care and service. However, it seems that referrals to occupational therapy are becoming lesser by day in specific settings and hospitals in Malaysia. This brings a perspective if other professions in the healthcare team understand the role of occupational therapists. This study is aimed at exploring the perception and misconceptions of allied health professionals on the roles of occupational therapists. A focus group discussion was conducted among seven allied health professionals using a heuristic approach. The interview data were analyzed thematically. Themes developed were (i) awareness of occupational therapy ecosystem, (ii) in cooperating togetherness for the benefit of the client, and (iii) addressing the limitation to enhance the occupational therapy visibility. Findings found that the knowledge of other healthcare practitioners and clients on the occupational therapist's role is still limited. Role confusion and overlapping are common among other allied health professionals. Occupational therapy needs to continually promote the profession's role and identity for the maximum benefit to the client and overall improvement.

## 1. Introduction

In rehabilitation, occupational therapy is an essential member of the interdisciplinary rehabilitation team alongside physiatry, physiotherapy, and speech-language pathology [1]. Occupational therapy has the most expansive coverage area of practice compared to other rehabilitation professions, from physical to mental health, from a clinical setting to community-based, from children to older people, and from people with disabilities to people with no disabilities. Effective interdisciplinary practices occur when each professional understands the role of one another [2]. This is beneficial as an optimal referral from the healthcare professionals will enable the client to receive all essential services. Occupational therapists play a vital role in the interdisciplinary team bringing a unique perspective to the client's care [3]. Working together as a team professional improves the client's outcome, increases the client's quality of care, and is cost-effective [4].

However, compared to other healthcare professions, awareness about occupational therapy is limited either in public or among healthcare professionals. In a mental health setting, Pottebaum and Svinarich [5] found that the relationship between knowledge on occupational therapy services and the number of referrals to occupational therapists is low. It was found that approximately 42% of psychiatrists were not fully aware of the services that occupational therapists towards mental health clients can provide. The services are vast and not limited to activities of daily living, cognition, and role change. This shows that psychiatrists' knowledge influences the number of referrals to occupational therapists. This study also reported that psychiatrists requested more education on the diverse skills and services that occupational therapy can offer to ensure that the maximum number of services towards clients can be given. Another study by Henderson et al. [6] also highlighted the perception of occupational therapists' role in community child and adolescent mental health services; limited knowledge of occupational therapy is still shown. In addition, medical and health science undergraduate students perceived occupational therapy as less recognized than other professions [7, 8]. Another research from Smith and Mackenzie [2] investigated the perception of a nurse and reported the need to increase communication, collaboration, and clarification about occupational therapy roles. This perception is also similar in that clients consider that the occupational therapy role is rarely known compared to other health practitioners [9]. Another research by Patel and Shriber [10] concluded that nurse practitioners need further education regarding occupational therapy roles and functions. Broad knowledge of occupational therapy was shown, but they lack confidence in what they know. A recent study by Alheresh and Nikopoulos [11] reported fair knowledge about occupational therapy among the rehabilitation team. The participants point out that the activities of daily living, hand therapy, and improving quality of life are the profession's primary responsibilities. Another research from Tariah et al. [12] reported that physiotherapists were more aware of occupational therapy than physicians and nurses. Healthcare practitioners need to take responsibility for understanding each other's roles and providing the necessary information and support for the client and caregivers [13].

Occupational therapy in Malaysia is facing challenges and limited recognition. Occupational therapy is relatively a small profession compared to other health professions and combined with low qualification requirements and low autonomy, and the occupational therapy profession is then facing marginalization [14]. Although healthcare practice in Malaysia has improved over the previous decades, it is predominated by hierarchy working and working in-silo culture [15, 16]. Hence, working with various disciplines was found to have a positive impact on clients in a holistic manner which is beyond than just related to the condition [17, 18]; for example, chronic pain clients not only received management on the pain but also had a better outcome on physical functions, mental health, emotion, and social and quality of life. Healthcare practitioners need to acknowledge the role of each other for the benefit of the client.

Nonetheless, this topic on awareness on occupational therapy from other parties received limited attention, and such a study is negligible in Malaysia. This indicates a requirement for such studies to be conducted. This study was aimed at exploring the perception and misconceptions of allied health professionals on the role of occupational therapy in a multidisciplinary team or general practice.

## 2. Materials and Methods

This study applied a heuristic approach to qualitative research, guiding data collection and analysis. The heuristic approach was used as participants, and researchers can learn from one another, reevaluating understanding of the issues discussed and how to overcome the issues [19, 20]. This study received ethical approval from the Research Ethics Committee of Universiti Kebangsaan Malaysia (NN-2019-006)

### 2.1. Participants, Recruitment, and Sampling

Participants were recruited via email or telephone. We employed criterion-based purposive sampling [21, 22]. Participants were invited to participate if they were (i) allied health professionals from various backgrounds, (ii) working in a clinical setting, and (iii) having experience working with occupational therapists for three years or more. We invited twelve (*n* = 12) participants, but only seven (*n* = 7) agreed to participate. Before the focus group discussion was conducted, the purposes of the study were explained in detail; an information sheet was given, and consent was obtained prior to agreeing to participate in the study. All participation is voluntary. Participants who agreed to participate in the focus group discussion were then contacted via email to inform the tentative date, time, and venue for the discussion.

### 2.2. Data Collection

A qualitative approach was chosen as the best method to answer questions related to experience, meaning, and perspective from the standpoint of the participants [20]. Data were collected via focus group discussion and was guided by Ivanoff and Hultberg's [19] framework. This includes three main themes, which are (i) focus-group research arena, the foundation and its core components; (ii) subjects, the role of the researcher and the participants; and (iii) activities, the specific tasks and procedures [19]. All participants were given the freedom to share their opinions and understanding about the issues discussed. Focus group discussion is the best way to exchange viewpoints and discuss disagreements between the multidisciplinary team and add diversity and enrichment of the discussion between participants [23]. The focus group discussion was conducted in one meeting room in a public institution, excluded from others to maintain anonymity and confidentiality of participants' participation in the study. A list of questions developed based on the review of the literature [2, 6, 10–12] was provided as guidance for the participants (Box 1) but they were asked not to follow prescriptively; the researcher has a role as a facilitator, to prompt the discussion and ensure that a comprehensive response was given for any unanswered question. The facilitator provides a fair opportunity for each participant to voice their opinions and enhance the discussion at any point deemed interested beyond the prepared question. The facilitator is assisted by another researcher who acts only as a note-taker. The focus group was conducted primarily in English; the participants were encouraged to discuss in English but were allowed to express their ideas in pidgin or in a formal Malay language to convey their ideas effectively as long as it is understandable by all members. The use of pidgin language is common in the Malaysian context, allowing for casual conversations. Casual conversations provide a robust and meaningful message and truly capture the participants' intention and expression; in other words, tacit and cultural understanding in the conservations is preserved [24]. Refreshments were provided throughout the discussion, and the participants were free to move and take at any time. Participants were also informed that they could withdraw from the study at any time without any consequences. The focus group discussion was held approximately one 1/4 hours, and all discussions were video and audio recorded with permission from all participants.

### 2.3. Data Analysis

This qualitative study's data management and analysis were guided by Sutton and Austin [23], which includes interpretation of data, transcribing and checking, reading between the lines, coding, and theming. Thematic content analysis was used to analyze the data. The audio recording will be listened to several times and transcribed verbatim while referring to the video recording by cross-referring the identity of the one speaking and analyzing for nonverbal cues. Listening to audio recording, video recording, and reading the transcript were done simultaneously to obtain the full expression of the participants. The technique called “reading between the lines” was implemented by hearing the participants' voice tone, emotional expression, and nonverbal cues to get a feel of the participants' experience and grasp the underlying message [23]. Codes were then generated based on the interview questions, and by identifying the issues, the participants revealed similarities and differences. Coding was conducted by making notes in the margin by naming sections of the text. Themes were then generated after drawing together codes from the transcript. The first and third authors did the coding and theme generation in a discussion session. The credibility of the findings was strengthened by conducting member-checking, providing opportunities for all participants to read, comment, and contribute to the findings. Data (coding) was sent to all participants via email to ensure the data's accuracy, fairness, and validity [25]. Trustworthiness of the findings was strengthened by sending the data (coding and themes) to the third author; this process is called confirmability, which requires inviting a member who was not involved in the focus group discussion to verify the synthesis of information conducted by the first author [25].

## 3. Results

The participant characteristics are presented in Table 1. The majority were female, and all were from different professions except physiotherapy, where two participated in the session. Five were from a public institution, and two were from a private higher education institution. Three main themes and six subthemes were generated after evaluating the transcript.

### 3.1. Theme 1: Awareness of Occupational Therapy Ecosystem

Although a broad definition of the role of occupational therapy cannot be expressed, most participants can identify some roles of occupational therapy based on what they observe, discuss, and experience while working together with occupational therapy.

#### 3.1.1. Exposure to Occupational Therapy

Most of the participants began to understand the role of occupational therapy better when they started working. This is due to their job duty which requires them to cooperate with occupational therapy as an interdisciplinary team. Some of the participants, especially those in the rehabilitation team, perceived that they had been exposed to the role of each other's profession during their study time.

*Participant A*: “We were taught if this type of case we should refer to occupational therapy, and for this type of case, we should refer to a physiotherapist. Thus we [rehabilitation team] of course know at least [each other's] role.” Although most of the participants have heard about occupational therapy during their study, they still lack an understanding of the role of occupational therapy. Some had no idea what occupational therapy interventions were before working with occupational therapists.

#### 3.1.2. Role of Occupational Therapy

Each allied health professional has their own opinions and views about the role of occupational therapy. Physiotherapists and speech therapists believe that their roles in occupational therapy are closely connected and give examples linked to their professions. This was noted by *participant E*: “I think our fields are very close to each other, so if we [speech] want to be successful in our intervention, we need occupational therapy to help.”

The perceptions of allied health professionals on the role of occupational therapy are mostly limited to the theory and the reason for referral. The role of occupational therapy was solely based on certain conditions. Some professionals refer to an occupational therapists primarily if they have patients with certain conditions. Each profession has certain cases that usually refer to occupational therapy.

*Participant E* shared, “I referred to occupational therapy due to attention issues which [is] also part of the sensory issue…sensory integration which I believe that is one of the fields that are under occupational therapy expertise, right?”

*Participant D* stated, “Normally, the case that I will refer to occupational therapy is for orientation mobility, usually due to vision drop or maybe [clients] who already have tunnel vision.”Some of the participants understood the role of occupational therapy in facilitating meaningful activity for the client.

*Participant E* stated that “For the adult cases, I understand that the occupational therapy role is for the hmm…training in Activities of Daily Living (ADL) part, right? Umm, they do handwriting training… teach the client how to do toilet training.”

*Participant F* on the other hand highlighted the role of occupational therapy in mental health settings on how to train the client to return to work program: “We [the psychologist] appreciate the role of occupational therapy in mental health settings, on how they [occupational therapy] train those with mental health problems to return back to their function in occupation…sewing nicely to be able to sell…train the client on how to do gardening, to be independent.”

Mostly, the participants believe that occupational therapy just implemented a similar technique with other professions but with a different aim. This was noted by *participant F*: “The technique used is the same, but the aim maybe is different. Occupational therapy used play therapy to focus on the occupation of the client, while we [clinical psychologists] used play therapy more on the psychological problem.” For those professions who rarely deal with occupational therapy, it was difficult to comprehend what is occupational therapy and it was unclear to identify the role. They considered occupational therapy as a huge and complex discipline as mentioned by *participant G* “…when I undergo occupational therapy, I still feel that occupational therapy and physiotherapy is similar. I cannot see the difference.” Another statement was made by *participant G*: “…and occupational therapy is actually huge, where any case can be referred to them [occupational therapy] since they [occupational therapy] can do anything.”

#### 3.1.3. Needs for Occupational Therapy

The perception of the importance of occupational therapy to the healthcare team and the client varies depending on the profession. Some participants realized the importance of occupational therapy and how they need each other in treating the same client. At the same time, some professions (i.e., nonrehab) believe that occupational therapy is not playing a significant role.

*Participant A*: “Each allied health professions have their function, and they [allied health professions] cooperate so they [allied health professions] are crucial for each other.” Some believed that clients would remove occupational therapy services first compared to other services on the client's list. However, some argued that occupational therapy would remain an important service compared to theirs.

*Participant F*: “I think we as professionals are fully aware of the importance of occupational therapy, but from what I experienced [sometimes] for a client, if there is a profession that they need to sacrifice, I think they [the client] will sacrifice occupational therapy first.” However, all participants believed that this situation would only happen when clients do not entirely understand the role of occupational therapy and due to a lack of explanation from the occupational therapy themselves.

*Participant E*: “When I asked did they [occupational therapy] explained why they [occupational therapy] did that [treatment]? [Clients said] no…So, usually when they [the client] say no, we [the speech therapists] will explain about [occupational therapy], and they will respond to it as they [the client] had never heard about it before…so that is one negative example, however, there are also a lot of positive comments too about [occupational therapy] ….”

### 3.2. Theme 2: In Cooperating Togetherness for the Benefit of the Client

#### 3.2.1. Interdisciplinary Working

Only a few participants, such as speech therapists and physiotherapists, frequently work with occupational therapy. They usually work together in a community setting, such as community-based rehabilitation centers and private hospitals. However, the number of active interdisciplinary working together is limited. Based on the participant's experience, the interdisciplinary working was mainly based on referral purposes either to or from occupational therapy and on a discussion regarding the client.

*Participant E*: “At the beginning of course referral, we (speech therapist) made a referral to occupational therapy, and I also went to the community-based rehabilitation centers to supervise the students.” One allied health professional stated that she had no chance of seeing the occupational therapy intervention. She needs to guess how occupational therapists work to convince the client before referring the client to the occupational therapy department. This was noted by *participant D*: “I do not know whether what I said was true. I said [to the client] that the therapy will teach you [client] on how to modify your [client] occupation at home...to cook…right? Umm, help you [the client] with how to manage your home…umm…the arrangement of your household…Is it right?”

### 3.3. Theme 3: Addressing the Limitation to Enhance the Occupational Therapy Visibility

#### 3.3.1. Restricted Viewable Job Scope

The participants see the limited understanding of the role of occupational therapy among the client and healthcare practitioners as a challenge. The clients sometimes have difficulties in recognizing occupational therapy purposes. According to one of the participants, this might be because the occupational therapy intervention resembles daily activities, where the therapeutic value is hindered, and tangible therapeutic adaptation in the activity is unnoticed.

*Participant F:* “Maybe the stereotype is easy to develop in occupational therapy activities because what you [occupational therapy] have been doing is very routine [in daily activities], right?”

Some participants believed that occupational therapy is well-known among those who only work directly with occupational therapy, such as a pediatrician, psychiatrist, neurologist, and orthopedic doctor. One participant agreed that this situation happened because the number of occupational therapists in the hospital is limited compared to physiotherapists.

*Participant C*: “If orthopedic neurologists, a pediatrician who is directly working with occupational therapy, they will [know about occupational therapy]. However, [for certain doctors] like ENT surgeons [maybe they do not know about occupational therapy.” Two participants highlighted the difficulty of arranging a time to work together with an occupational therapist as a challenge. One participant who has worked with occupational therapy for 12 years shared her experience with difficulty setting time to discuss about a client.

*Participant E*: “We sometimes make a full discussion when we meet up about our clients and normally because of timing, we cannot find time to sit together, so we will only discuss our clients through the phone.” One participant shared that she was keen to observe and know more about occupational therapy treatment but was not given a chance to set time with the occupational therapy itself. This was noted by *participant D*: “The most important thing is that we [allied health professionals] have to understand each other's role…I want to understand but were not given a chance to [understand].” Some professions such as physiotherapists and clinical psychologists showed confusion on occupational therapy due to role overlapping.

*Participant F*: “I am always confused about the role of clinical psychologists and occupational therapy in the mental health field and also in neurodevelopmental disorders in pediatrics.”

*Participant C*: “Clients tend to get confused when doing exercise, it seems similar especially when it involves hand fracture…so I think the way of working is sort of the same but [allied health professionals] have to focus more in terms of explanation about the aims.” Besides, the all-rounding capability of occupational therapy services might develop a problem such as limited sharing of referrals to other practitioners. Participants also complain that it is difficult to frequently get the client's referrals to occupational therapy, especially in the government hospitals.

#### 3.3.2. The Intangibility of Occupational Therapy Identity

Participants indicated that many clients and the public did not understand the role of occupational therapy. Two participants stated no knowledge about occupational therapy outside the healthcare sector. This was noted by *participant F*: “Sometimes when I asked do they [the client] be referred to occupational therapy? [Clients will ask] What is occupational therapy?” The participants expressed strong views about the importance of practicing client-centeredness for the long-term effect of the client. They emphasized the need for occupational therapy to explain the reason or aim of the services for the client to understand. This will ensure that the client will see that the occupational therapy treatment is as necessary as others.

*Participant C*: “In the private sector, they need to pay a lot to come for the treatment session, so we need to emphasize the importance [of the treatment] for them [client] to come… that is why we always emphasized the student itself, before doing the assessment, we need to explain why we did that [the assessment].” The participants believed that promoting occupational therapy requires explicit and intensive promotion such as seminar workshops or training. One participant said one of the ways to improve the public and client's understanding about occupational therapy is by establishing a course on effective communication for occupational therapy to give clinical reasoning on the services provided to the client.

*Participant E*: “Faculty of Health Sciences will establish a course about effective communication towards the client, so I think it is essential. A clinician has to know how to explain what they are doing and the reason they did it [the treatment], so the client will not question about the importance of our treatment.”

*Participant F*: “Based on my experience, the reason I understand more about occupational therapy is that my friend who is an occupational therapy always teaches me on how to use the [occupational therapy] knowledge to help me.”

The participants were also asked about having a uniform; however, all participants disagreed with this idea. The participants deemed having a uniform as implying inferiority in professional identity.

*Participant A*: “No, there is no value in wearing a uniform as we will get confused about which profession is which.” This was also supported by *participant E*: “I do not think it [uniform] is necessary, however, if there is a world occupational therapy day to celebrate then you can wear it [uniform] during that time, but for working, sorry, I do not think it is needed.”

## 4. Discussion

This study indicates that healthcare professionals have a certain degree of awareness about the role of occupational therapy, however, limited to the exposure available. The professions under the rehabilitation team, such as speech therapy, audiology, and physiotherapy, have more understanding of the role of occupational therapy. Research from Tariah et al. [12] also concludes that the profession under the rehabilitation team, such as physiotherapists, has better knowledge about occupational therapy than physicians and nurses. However, some allied health professionals such as clinical psychologists and physiotherapists seem to have role confusion in certain aspects. The issue of role overlapping is expected in the literature where the intervention activity resembles each other [26, 27]. A simple argument, even mentioned by the participants, is that each discipline has different intervention goals even doing the same treatment. However, this is partially true as each discipline approaches the intervention differently based on clinical reasoning and intensity of the therapeutic value, although it looked similar by the lay eye. The activity has been tailored and modified to excavate the optimal therapeutic value at the microlevel. In addition, occupational therapy should strengthen the practice by returning to the essence of the profession's philosophy by using occupation as a means and as an end [28]. This will develop a more distinctive role of occupational therapy than the others.

One aspect notified is other professions reflecting the occupational therapy role from the lens of their discipline. They are compared with their preexisting knowledge with the practice known as schemata [29]. The absence, incomplete, or fragmented knowledge about other disciplines is not helpful to make other disciplines understand the values, roles, and contributions that occupational therapy can make. Interprofessional education touching each health profession discipline, philosophy, and general role should be formally made in the academic curriculum and continuous professional education to expose and reshape the schemata of each healthcare individual [29]. This aspect is critical as healthcare practices are now moving towards team-based and collaborative working, which is more beneficial for the clients [30, 31]. In allied health education, exposure to other professions is lacking and only focuses on the own discipline.

Most of the participants highlighted the importance of interdisciplinary working; however, the findings of this study showed that the interdisciplinary working between health professions is limited; only at the basic level of the referral phase. This study shows the issue is pertinent where allied health practices usually work in a silo with little opportunity for crosscommunication due to the distinct nature of working. More active collaboration working is required among healthcare practitioners. Active collaboration in interdisciplinary working such as intervening with the client simultaneously at the same time and setting is helpful to reduce role overlapping, improving communication cost and saving time [32, 33]. Active collaboration is among the best methods to promote the profession to other professions as close, direct, and frequent experience contributes towards understanding [32]. Interprofessional working should be encouraged either in education or practice.

Occupational therapists have a significant responsibility in making their profession visible. The client might have difficulty differentiating occupational therapy roles because the intervention seems “normal” as their daily activities. This makes health professionals, clients, and careers often understand the role of many health professionals but have difficulty understanding occupational therapy's role [2]. Only medical practitioners and nurses are acknowledged in the healthcare industry, while other allied health professions are unnoticed. This study is supported by a study by Darawsheh [9]. However, studies [34] with occupational therapists were found to perceive otherwise, where the occupational therapy profession is known by others [16, 34]. This situation occurs because occupational therapists may have a misperception of overestimating the profession's awareness. The critical element is that occupational therapists should apply a client-centered approach and constant client education to enhance the client's awareness of the profession. Having dedication on own work only is not enough where the occupational therapist needs to be proactive and extrovert in conveying, clarifying, and promoting the profession's identity. Client education is essential for therapists to explain the benefit of each intervention [35]. Occupational therapists need to ensure that the client's message and reasoning for the service are adequately understood. Lack of understanding by the clients and other colleagues is harmful to the profession, consequent to the reduction of referral and career opportunities, and makes the service vulnerable for abandonment due to misperception on the lack of importance. Occupational therapists need to take the responsibility to promote their profession proactively.

It is appalling that uniform is less desired among the participants. Uniform is a simple yet effective solution to make the profession noticeable and increase visibility [36]. However, there is a sentiment against uniform wearing, where it imposes lower status and professional titles than medical practitioners (i.e., medical doctors) [36, 37]. Moreover, the ignorance of medical practitioners to be aware of the specific professions according to the uniform (i.e., generalized all uniform individuals as nurses) has offended the individuals [37, 38]. However, relying only on verbal explanation in differentiating roles and defending professional identity is effort consuming, short-term, and less effective (i.e., easily forget). Therefore, proper visual identification should be made available, such as having a sizeable badge-wearing uniform is fairly made to all health professions, including medical practitioners.

## 5. Study Limitations and Recommendations

A small sample size of the study cannot be generalized to all Malaysian populations. Hence, the participants were mostly in one locality and were working at the higher education institution, providing limited scope and homogeneous exposure. In addition, this study did not include rehabilitation doctors and nurses in a hospital setting. Due to budget, setting, and time constraints, we cannot increase our effort.

Future research should be conducted in a different setting such as in the government and private hospital, community, and industrial settings and include other professionals such as a doctor, nurse, social worker, and case manager to provide a richer understanding of the perceptions and experiences on the role of occupational therapy. In our study, we encountered challenges to involve a wider setting due to unforeseen circumstances. Another limitation includes the researchers themselves being occupational therapists which may lead to bias in the data interpretation. However, we believe the impact is minimal as trustworthiness has been conducted where the participants have validated the study findings.

## 6. Conclusions

These study participants provided valuable experiences and perceptions on how occupational therapy is perceived among allied health professionals, clients, and the public. Although occupational therapists are perceived as essential members of an interdisciplinary team, the knowledge of other allied health practitioners and clients on the role of occupational therapy is still limited. Therefore, it is essential for occupational therapy to continuously promote the profession's role and identity for the maximum benefit to the client and overall improvement.

## Figures and Tables

**Box 1:**
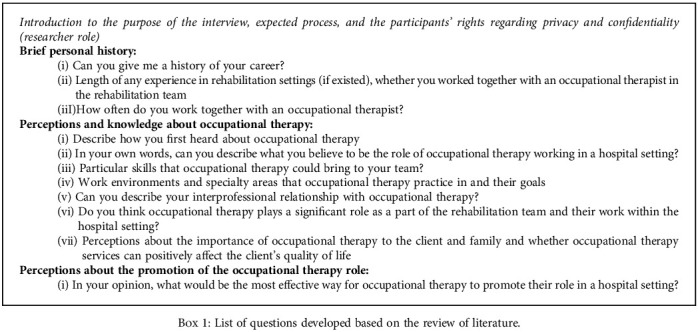
List of questions developed based on the review of literature.

**Table 1:** Characters of the participants (*N* = 7).

Participant	Age	Gender	Race	Education	Profession	Duration of work experience	Duration working with occupational therapists
A	36	Female	Malay	Master	Audiologist	12 years	11 years
B	32	Female	Malay	Master	Physiotherapist	3 years	3 years
C	35	Female	Malay	Master	Physiotherapist	10 years	10 years
D	31	Female	Malay	Bachelor's degree	Optometrist	9 years	7 years
E	35	Female	Malay	Bachelor's degree	Speech therapist	12 years	12 years
F	47	Male	Chinese	Master	Clinical psychologist	16 years	16 years
G	43	Female	Malay	Diploma	Medical lab technologist	18 years	3 years
